# Untargeted metabolomics reveals distinct metabolic profiles and putative bioactive metabolites in cell-free supernatants of food-grade microorganisms

**DOI:** 10.3389/fmicb.2026.1854827

**Published:** 2026-10-02

**Authors:** Denise Drago, Luca Marini, Eleonora Bafile, Federica Federici, Radmila Pavlovic, Marynka Ulaszewska, Gianfranco Frigerio, Laura Manna, Annapaola Andolfo

**Affiliations:** 1 Proteomics and Metabolomics Facility (ProMeFa), Center for Omics Sciences (COSR), IRCCS San Raffaele Scientific Institute, Milan, Italy; 2 NTP Biotech S.r.l., Teramo, Italy; 3 PNK Farmaceutici S.p.A., Teramo, Italy

**Keywords:** untargeted metabolomics, postbiotics, by-product waste, probiotics, industrial fermentation

## Abstract

Over the past decade, the understanding of the role of microorganisms in health and disease has expanded considerably, with many of the beneficial effects traditionally attributed to probiotics now also being associated with their metabolites, collectively referred to as postbiotics. In this context, postbiotics are increasingly being explored as complementary components of broader microbiome-centered strategies. Fermentation supernatants are commonly regarded as industrial by-products; however, they contain a diverse repertoire of microbially derived metabolites with potential biological relevance. The valorization of these fermentation streams therefore represents a promising strategy for identifying novel compounds capable of modulating host physiology. Five food-grade microorganisms-*Lactobacillus amylovorus* SGL 14, *Bifidobacterium breve* SGB 01, *Lentilactobacillus kefiri* SGL 13, *Lactiplantibacillus plantarum* SGL 07, and *Ligilactobacillus salivarius* SGL 03-were cultured under standardized conditions, and their cell-free supernatants were characterized using untargeted metabolomics to identify differential metabolic profiles. Each strain displayed a distinctive metabolomic signature enriched in metabolites previously associated in the literature with potential biological activities. Collectively, the identified compounds have been linked to antimicrobial, immunomodulatory, neuroactive, antioxidant, epithelial-supportive, and metabolic functions. However, the present study did not include direct functional validation of these activities, and the proposed biological relevance of the detected metabolites should therefore be considered putative and inferred from previous literature evidence. Overall, untargeted metabolomics/lipidomics revealed that fermentation media derived from food-grade microorganisms represent a valuable and underexplored source of multifunctional metabolites with potential postbiotic applications. These findings support the concept of valorizing microbial fermentation by-products as sustainable reservoirs of biologically relevant molecules within a circular bioeconomy framework. In industrial settings, fermentation broths traditionally considered waste streams may be recovered and repurposed, potentially reducing disposal costs and environmental impact. Furthermore, optimization of fermentation processes prior to large-scale metabolite production will be essential to maximize yield, improve process efficiency, and support their future development for nutraceutical and health-related applications.

## 1 Introduction

In the last decade, our understanding of the role of microorganisms in health and disease has evolved considerably. Early efforts largely centered on using live probiotics to alter the gut microbiota in order to promote health benefits (Amara and Shibl, 2015; Kerry et al., 2018; Plaza-Diaz et al., 2019; Zommiti et al., 2020). However, accumulating scientific evidence suggests that some of the beneficial effects traditionally associated with probiotics may also be mediated by microbial metabolites and non-viable microbial components (Taverniti and Taverniti, 2011; Aguilar-Toalá et al., 2018). These bioactive compounds, recently referred to as postbiotics, are increasingly investigated for their potential role in modulating host-related functions (Scott et al., 2022).

The term “postbiotic” is the subject of ongoing debate among scientists, with multiple suggested definitions since it was for the first time described in 2013 as “any factor resulting from the metabolic activity of a probiotic or any released molecule capable of conferring beneficial effects to the host in a direct or indirect way” (Tsilingiri and Rescigno, 2013). Many later definitions, consistent with this perspective, emphasized microbial metabolites or secreted bioactive compounds (Johnson et al., 2019; Wegh et al., 2019). Others have also included non-viable microbial cells and/or their components (Salminen et al., 2021; Collado et al., 2019). More recent conceptual and regulatory frameworks have further expanded the broader postbiotic category to include cell-free fermentation supernatants and related fermentation-derived preparations (Guglielmetti et al., 2025).

Over the years, scientific interest has increasingly shifted toward bioactive compounds generated during microbial fermentation, including short-chain fatty acids, enzymes, peptides, amino acids, polyamines, and exopolysaccharides, many of which have been associated with potential health-related functions (Liang and Xing, 2023; Prajapati et al., 2023; Asefa et al., 2025).

Since postbiotics do not contain viable microorganisms, they may offer advantages in terms of safety and stability, contributing to growing interest in their potential health-related applications (Abdul Hakim et al., 2023). Cell-free supernatants (CFS), obtained by the centrifugation of microbial cultures, are a mixture of “soluble factors secreted by living bacteria or released by bacterial lysis” (Martín and Langella, 2019). These supernatants are recognized as promising preparations containing putative postbiotic metabolites due to their distinct metabolic fingerprints and bioactive properties (Kumar et al., 2024; Aguilar-Toalá et al., 2021).

Advances in microbiome research, particularly through the integration of untargeted metabolomics, have enabled a comprehensive characterization of these metabolites. This approach allows for the identification of both known and previously uncharacterized compounds, uncovering the molecular complexity and diversity present in the CFS and providing deeper insights into host–microbe interactions and the functional potential of microbial-derived products.

Metabolomic and lipidomic analyses of probiotic strains, particularly from the *Lactobacillus* genus, have attracted growing attention in both food industry -where these bacteria are widely used as starter cultures in fermented products- and in medical research, due to the identification of health promoting compounds generated during fermentation (Spaggiari et al., 2024; Chamberlain et al., 2019; Scano et al., 2021).

In parallel, increasing attention has been directed toward the valorization of fermentation by-products within a circular bioeconomy framework. Fermentation broths, once considered waste, are now recognized as valuable sources of metabolites with potential applications not only in nutraceuticals, but also in fields such as agriculture, animal nutrition, and environmental sustainability, contributing to more sustainable production processes.

In this study, we investigate the metabolite composition of CFS obtained from five food-grade bacteria strains using an untargeted metabolomic and lipidomic approach. The aim is to identify distinct metabolite profiles and explore their potential as value-added nutraceutical ingredients, while supporting more sustainable and resource-efficient production strategies.

By integrating advanced analytical technologies with optimized fermentation processes with a strong focus on both economic and ecological sustainability, this approach offers a holistic strategy to meet market demands while addressing global environmental challenges.

## 2 Materials and methods

### 2.1 Bacteria and growth conditions

#### 2.1.1 Strains and culture conditions

Five different bacteria were selected for this study: *Ligilactobacillus salivarius* SGL 03 (DSM 25381), *Lactiplantibacillus plantarum* SGL 07 (DSM 25767), *Lentilactobacillus kefiri* SGL 13 (DSM 27331), *Lactobacillus amylovorus* SGL 14 (DSM 27014) and *Bifidobacterium breve* SGB 01 (DSM 25769). The strains were selected from the NTP Biotech Srl biobank (Castelnuovo Vomano, Teramo, Italy) and have been previously investigated in different studies for their functional properties and potential health-related applications (Brandi et al., 2019; Brandi et al., 2020; Pidutti et al., 2018; Manna et al., 2020; Manna et al., 2023; Panebianco et al., 2021; Panebianco et al., 2023; Giancola et al., 2024), supporting their relevance for further metabolomic characterization. The bacterial strains, were isolated from different sources: SGL 03, SGL 07, and SGB 01 from healthy human stools, SGL 14 from healthy animal stools, and SGL 13 from fermented milk. The isolates were cryopreserved in MRS broth (BD Difco TM) added with 0.05% N-acetylcysteine (Sigma Aldrich) and 15% glycerol. The strains were revitalized from frozen stock on MRS agar plates prepared with MRS broth (BD Difco TM) added with 0.05% N-acetylcysteine (Sigma Aldrich) and 1.5% agar (Liofilchem). The plates were incubated at 37 °C for 24–48 h in an anaerobic jar using AnaeroGen sachets (Thermo Fisher Scientific). In order to reactivate microbial metabolism, a single colony of each strain was inoculated into 10 mL tubes of MRS broth supplemented with 0.05% N-acetylcysteine.

#### 2.1.2 Culture media and fermentation conditions

Fermentation processes were carried out in 2 L bioreactors (Omnitec Srl, Milan, Italy) operating in discontinuous mode (batch) using 3 different media depending on the strain. *Ligilactobacillus salivarius* SGL 03 (MIC_LAC_SALI), *Lactiplantibacillus plantarum* SGL 07 (MIC_LAC_PLAN) and *Bifidobacterium breve* SGB 01 (MIC_BIFI_BREV) were cultured in M1 medium containing 10 g/L primary yeast peptone, 10 g/L hydrolysed wheat protein, 5 g/L yeast extract, 5 g/L sodium acetate trihydrate, 2 g/L potassium phosphate dibasic, 2 g/L ammonium citrate dibasic, 0.1 g/L magnesium sulfate heptahydrate, 0.05 g/L manganese sulfate monohydrate, 0.5 g/L N-acetylcysteine, 60 g/L glucose, 10 g/L polysorbate 80.

*Lactobacillus amylovorus* SGL 14 (MIC_LAC_AMYL) was cultured in M2 medium containing 18 g/L meat peptone, 4 g/L yeast extract, 2 g/L potassium phosphate dibasic, 2 g/L ammonium citrate dibasic, 0.2 g/L magnesium sulfate heptahydrate, 0.05 g/L manganese sulfate monohydrate, 0.5 g/L N-acetylcysteine, 60 g/L glucose, 10 g/L polysorbate 80, 0.67 g/L sodium oxalate.

*Lentilactobacillus kefiri* SGL 13 (MIC_LAC_KEFI) was cultured in M3 medium containing 14 g/L primary yeast peptone, 25 g/L yeast extract, 5 g/L sodium acetate trihydrate, 2 g/L potassium phosphate dibasic, 2 g/L ammonium citrate dibasic, 0.1 g/L magnesium sulfate heptahydrate, 0.05 g/L manganese sulfate monohydrate, 0.5 g/L N-acetylcysteine, 60 g/L Glucose, 10 g/L polysorbate 80.

To enhance the yield of target metabolites, the basal medium M1 was tested both without supplementation and with the addition of two different concentrations of the aromatic amino acids tryptophan and phenylalanine. Specifically, two supplementation levels were used: a lower concentration with 0.5 g/L tryptophan and 1.5 g/L phenylalanine, and a higher concentration with 1.5 g/L tryptophan and 3.0 g/L phenylalanine. Yield optimization experiments were conducted exclusively with SGL 07 used as reference organism.

Bioreactors containing fermentation media were sterilized at 105 °C for 90 min and then hermetically sealed to preserve anaerobic conditions. No gas insufflation was applied. The bioreactors were inoculated with 1.5% (v/v) of an exponential-phase pre-culture, previously grown in the same medium. The pH was continuously monitored with a Hamilton pH meter (Hamilton Bonaduz, Switzerland) and maintained at 5.5–5.7 by automatic addition of 5 M NaOH. Absence of dissolved oxygen was monitored via an inline oxygen sensor (B&C Electronics). Temperature was maintained at 30 °C for SGL 13 and 37 °C for all the other strains. The stirring speed was maintained within a range of 100 to 200 rpm. Cell growth was monitored by measuring the optical density at 600 nm (OD₆₀₀) and by determining the colony forming unit CFU/mL with plate count method. At the end of each fermentation, 100 mL of culture was harvested and centrifuged at 5,000 × g for 15 min to remove cells, yielding the CFS. The CFS was then sterile-filtered through a 0.2 μm syringe filter and stored in aliquots at −80 °C for subsequent untargeted metabolomic analysis and HPLC quantification. All fermentations were performed in duplicate, providing two independent biological replicates for each strain.

### 2.2 Untargeted metabolomic and lipidomic analysis

#### 2.2.1 Metabolite extraction

The CFS, containing the extracellular metabolites, and corresponding blanks were normalized by adding an appropriate volume of MilliQ water prior to performing untargeted metabolomics analysis. In this manner, all the examined broth samples correspond to 3×10^5^ CFU/mL (Supplementary Table S1). The metabolite extraction was conducted by adding 75 μL of cold extraction mixture (acetonitrile: H_2_O = 2:3, v/v) to the 25 μL of each broth sample, followed by incubation at 4 °C for 15 min under gentle agitation (600 rpm) and then centrifugation at 16,000 rcf for 15 min at 4 °C. Quality control (QC) samples were obtained by mixing equal amounts of each sample.

#### 2.2.2 Liquid chromatography mass spectrometry (LC–MS/MS) data acquisition

The resulting supernatants were subsequently utilized for untargeted metabolomics and lipidomics analysis by using Triple TOF 5600+ mass spectrometer (SCIEX) coupled to UPLC 1290 (Agilent Technologies). Chromatographic separations were carried out independently on three complementary stationary phases: hydrophilic interaction liquid chromatography (HILIC, BEH AMIDE (1.7 μm, 2.1 × 150 mm), Waters), reverse-phase (RP) chromatography (HSS T3 C18 (1.8 μm, 2.1 × 100 mm, Waters) for metabolomics, and BEH C8 (1.7 μm, 2.1 × 100 mm), Waters) for lipidomics. This dual approach (HILIC and RP) for metabolomics enhances the coverage of analytes with diverse physicochemical properties. For the BEH AMIDE and RP-C18 runs, supernatants were directly injected into the LC–MS/MS system. On the other hand, for the BEH RP-C8 analysis, the second aliquot was brought to dryness under a nitrogen stream and reconstituted in 100 μL of isopropanol/methanol 1:1, v/v mixture before injection. This targeted drying and organic reconstitution strategy, combined with the selective retention behavior of the liquid chromatography columns, effectively prevented the carryover and analysis of high-molecular-weight hydrophilic culture media components and surfactants, such as polysorbate 80, into the final evaluated lipid fraction. Each biological replicate was analyzed in technical triplicate, injecting 10 μL in negative acquisition mode and 5 μL in positive acquisition mode for RP-C18 and BEH RP-C8 analyses. For BEH AMIDE column, only positive modality was acquired by injecting 10 μL of each sample. Chromatographic conditions were based on the method previously published by (Want et al., 2013), with minor modifications. Briefly, for BEH AMIDE column, the flow rate was set at 0.6 mL/min with a gradient of solvent A (acetonitrile, 0.1% formic acid) and solvent B (water, 0.1% formic acid). The gradient started at 2% B; increased to 60% B in 10 min; held constant at 60% B for 12 min, and returned to 2% B in 0.5 min. The column was maintained at 40 °C while the samples were at 4 °C. For the RP-C18 column, the flow rate was set at 0.6 mL/min with a gradient of solvent A (water, 0.1% formic acid) and solvent B (acetonitrile, 0.1% formic acid). The gradient started at 2% B; increased to 95% B in 14 min; held constant at 95% B for 5 min, and returned to 2% B in 0.1 min. The column was maintained at 50 °C while the samples were at 4 °C. The flow rate for BEH RP-C8 was 0.4 mL/min with a gradient of solvent A (water, 0.1% formic acid) and solvent B (methanol/isopropanol = 85/15, 0.1% formic acid). The gradient started at 75% B, increased to 85% B in 2 min and then to 99.9% B in 12 min; held constant at 99.9% B for 5 min and returned to 75% B in 1 min. Again, the column was kept at 50 °C while the samples were at 4 °C. The gradient for each column remained identical regardless of the acquisition mode used. The mass spectrometer was operated in both polarities by acquiring data in SWATH mode. For BEH AMIDE, the mass range was 50–500 *m/z*, the acquisition cycle was 0.9 s, including 10 SWATH windows, each of 45 Da. For RP-C18 analysis, the mass range was 50–850 *m/z*, the acquisition cycle was 1.15 s, including 16 SWATH windows, each of 50 Da. For the BEH RP-C8 analysis, the mass range was 150–1,200 *m/z*, the acquisition cycle lasted 1.95 s, including 24 SWATH windows each of 45 Da. Ionization source parameters for both polarities were the same for BEH AMIDE, RP-C18 and BEH RP-C8 workflows: ion spray voltage floating (ISVF) 5,500 V (−4,500 V); declustering potential (DP): 80 V (−80 V); collision energy (CE): 10 V (−10 V); temperature 500 °C; curtain gas, 35 psi and ion source gas GS1: 33 psi and GS2: 58 psi, respectively. For fragmentation, the collision energy and its spread (CE ± CES) were 35 ± 15 V and −35 ± 15 V for positive and negative ionization modes, respectively, for BEH AMIDE (only positive mode) and RP-C18 method; 45 ± 15 V and −45 ± 15 V for positive and negative ionization modes for BEH RP-C8 method. Automated calibration was performed using an external calibrator delivery system (CDS) that infuses APCI positive or negative calibration solution every 5 sample injections. All samples were analyzed in a completely randomized manner with QC injection every 8 running samples. Pooled QC samples were injected throughout the analytical sequence to monitor overall analytical consistency, while sample randomization and instrument calibration were used to minimize potential run-order and instrumental effects, in accordance with established principles of quality assurance in metabolomics (Kirwan et al., 2025).

#### 2.2.3 Data processing and statistical analysis

The .wiff files acquired by both workflows (HILIC and RP) were converted to .abf by using Reifycs Abf Converter^1^ and analyzed with MS-DIAL (version 4.70) (Tsugawa et al., 2015) for peak picking, gap filling, alignment, deconvolution and metabolite annotation separately for negative and positive ionization modes. The metabolite identification was performed using MS/MS Positive Public library v. 14.0 and MS/MS Negative Public library v. 14.0. The MS-DIAL output was manually inspected to verify the annotation of the metabolites. The LOWESS method, available within MS-DIAL software, was used for the normalization. Lipidomic data processing was performed using the MS-DIAL software (v.4.92) within its dedicated lipidomics workflow. Lipid annotation was based on the accurate *m/z* of pseudomolecular ions (or adducts), MS/MS fragmentation data and acceptable retention times, employing a hybrid scoring system that integrates classical spectral matching with fragmentation rules specific to each lipid subclass. Lipid identification was conducted using the LipidBlast library integrated into MS-DIAL, which enables annotation of both lipid classes and individual species based on MS/MS data. When necessary, manual structure elucidation was carried out to complement automated assignments. All datasets, containing the normalized area, were saved as .txt by MS-DIAL. Subsequent data analysis was performed in R, using the tidyverse (Wickham et al., 2019), margheRita (Mosca et al., 2024), and GetFeatistics (Frigerio, 2025) packages. According to the guidelines set by the Metabolomics Standards Initiative (MSI) (Canzler, 2021) the identification of metabolites is classified into levels of confidence with some modifications reported in margheRita R package (Mosca et al., 2024). The levels indicate the degree of confidence and reliability in molecule identification. Level 1 is supported by retention time (RT), *m/z* and MS/MS fragmentation pattern. It requires comparison with an authentic chemical standard analyzed under identical experimental conditions and it is considered the highest and definitive metabolite identification level. Level 2 is supported by *m/z* and MS/MS fragmentation pattern with no matching for RT. It can also be based on spectral similarity to public or commercial spectral libraries and indicates a putative identification. Level 3 is supported by RT and *m/z* without any information for the MS/MS fragmentation pattern. In this case structural information is partial and the exact compound is not known. MS data may suggest a likely class or substructure, but not a specific molecule and indicates a tentative identification (Theodoridis et al., 2026). Analyses were run separately for the BEH_POS, RP-C18_NEG, and RP-C18_POS, RP-C8_NEG, and RP-C8_POS datasets. For each dataset, features were first blank-subtracted: for every feature, the mean intensity in the associated blank samples was subtracted from the sample intensity. Following the margheRita workflow, features were then normalized using probabilistic quotient normalization (PQN) with pooled quality-control (QC) samples as the reference, to reduce between-sample variation arising from differences in overall sample concentration and global signal intensity while preserving relative feature-specific differences. To prioritize features with biological variability over technical variability, we computed, for each feature, the ratio of the coefficient of variation (CV; standard deviation divided by the mean) in study samples to the CV in pooled QC samples, and retained only features with a CV ratio (samples/QC) greater than 2. Principal component analysis (PCA) was used for data visualization after pareto scaling (mean-centering and division by the square root of the standard deviation).

Differences among the five strain groups were assessed by one-way ANOVA, with all observations including technical and biological replicates, followed by Tukey’s Honestly Significant Difference (HSD) test for pairwise comparisons. Given the large number of features, *p*-values of both the global and pairwise comparison were adjusted for multiple testing using the false discovery rate (FDR). Adjusted *p*-values below 0.05 were considered statistically significant. Feature annotation and identification was performed using the margheRita libraries (Mosca et al., 2024).

After processing BEH_POS, RP-C18_NEG, and RP-C18_POS separately, results were merged across the three modes; when the same metabolite was detected in more than one mode, only the instance with the highest mean intensity was retained. Chemical identifiers (PubChem CIDs) were mapped to KEGG compound IDs using metaboliteIDmapping (Canzler, 2021). Pathway enrichment was performed with FELLA (Picart-Armada et al., 2018), and graphs were generated with igraph (Csárdi, 2025). KEGG organism codes were: “has” (*Homo sapiens*); “lam,” “lai,” “lay” for *L. amylovorus* SGL14; “lkf” for the *L. kefiri* SGL13 strain; “lsl,” “lsi,” “lsj” for *L. salivarius* SGL03; “lpl,” “lpj,” “lpt,” “lps,” “lpr,” “lpz,” “lpb,” “lpx” for *L. plantarum* SGL07; and “bbv,” “bbru,” “bbre,” “bbrv,” “bbrj,” “bbrc,” “bbrn,” “bbrs,” “bbrd” for *B. breve* SGB01. For enrichment analysis using the *Homo sapiens* organism code, we included all significant metabolites; for each strain-distinct analysis, we included only metabolites that were significantly different in at least one pairwise comparison involving that strain. Finally, all the codes written to produce the data elaboration described in this section are available in a public GitHub repository (Frigerio et al., 2025).

Finally, identified lipids and their ontology were subjected to downstream statistical and pathway analysis using the same R scripts applied for metabolomics data.

### 2.3 Quantification of 3-phenyllactic acid (PLA) and indole 3-lactic acid (ILA)

#### 2.3.1 Targeted metabolomics analysis by LC–MS/MS

Targeted metabolomics analysis was applied to all broth samples by following the same metabolite extraction procedure presented in Section 2.2.1. Quantifications were obtained by comparing samples with standard calibration curves. Deuterium-labeled standards, 3-Phenyllactic acid-d_3_ and Indole-3-lactic acid-d_5_ (Toronto Research Chemicals) were added to all samples and included in the calibration curve of unlabelled PLA and ILA, to correct for metabolite extraction yield. The mass spectrometer used for targeted approach was the same used for untargeted analysis as previously described. Only RP-C18 chromatography with HSS T3 C18 (1.8 μm, 2.1 × 100 mm, Waters) column was applied to quantify PLA and ILA, using the same chromatographic conditions as above reported. The mass spectrometer was operated only in negative polarity by acquiring data in Product Ion mode (PI) to detect PLA and ILA and their specific fragments (PLA, parent ion (M-H)^−^ 165.05 and fragment ion (M-H)^−^ 119.05; ILA, parent ion (M-H)^−^ 204.06 and fragment ion (M-H)^−^ 130.06). The calibration curve in blank samples (to mimic the matrix effect in the samples) for PLA included 0.5 ng/μL, 1 ng/μL, 7.5 ng/μL, 15 ng/μL, 25 ng/μL while the calibration curve for ILA included 0.05 ng/μL, 0.1 ng/μL, 0.75 ng/μL, 1.5 ng/μL and 2.5 ng/μL (Supplementary Figure S1).

#### 2.3.2 RP-HPLC/dad

The CFS, filtered with a sterile 0.22 μm filter, was diluted in water and analyzed by using reverse-phase high performance liquid chromatography (RP-HPLC) equipped with a Kinetex C18 column (150 mm × 3.0 mm, 5 μm, Phenomenex). Both PLA and ILA were analyzed with a DAD detector (Agilent Technologies) at 210 nm. Elution was performed with acetic acid 0.1% in water and acetonitrile according to the procedure described by Jung et al. (2019). Quantifications were obtained by comparing samples with standard calibration curves.

## 3 Results

### 3.1 Fermentation of *Lactobacillus* and *Bifidobacterium* strains for broth recovery

The fermentation process was terminated at strain-distinct time points corresponding to the optimal growth phase: 7 h for *Ligilactobacillus salivarius* SGL 03, 15 h for *Lactobacillus amylovorus* SGL 14, 40 h for *Lentilactobacillus kefiri* SGL 13, 20 h for *Lactiplantibacillus plantarum* SGL 07, and 24 h for *Bifidobacterium breve* SGB 01.

Cell growth, monitored by OD₆₀₀ and confirmed by viable counts, reached final titers of approximately 5 × 10^9^ CFU/mL for *L. salivarius* SGL 03, 9 × 10^8^ CFU/mL for *L. amylovorus* SGL 14, 2 × 10^9^ CFU/mL for *L. kefiri* SGL 13 and *L. plantarum* SGL 07, and 3 × 10^9^ CFU/mL for *B. breve* SGB 01.

At the established endpoints, fermentation broths were recovered and processed to obtain the CFS. These samples were subsequently employed for untargeted metabolomic analysis aimed at identifying putative bioactive compounds.

### 3.2 Identification of bioactive compounds in fermentation broths through metabolomic analysis

#### 3.2.1 Untargeted metabolomics analysis

Untargeted metabolomics analysis of fermented CFS was employed as an analytical approach to identify molecules secreted by individual probiotic strains during fermentation processes, with the aim of interpreting these compounds in terms of their potential nutritional and/or physiological activities of commercial relevance. Metabolomic profiling of fermentation broths was obtained by applying high-resolution mass spectrometry (HR-MS) coupled with two complementary chromatographic separation techniques: HILIC, BEH AMIDE and RP-C18 (Hajnajafi and Iqbal, 2025). The BEH AMIDE column enabled the enhanced separation of highly polar compounds, including amino acids and their metabolites, sugars, and small organic acids. In contrast, the RP-C18 column was employed to efficiently retain and resolve moderately polar metabolites such as drugs, steroid derivatives, and bile acids. Quality-control (QC) processing enabled the reduction of sample-wide intensity differences through PQN normalization and the removal of features showing excessive analytical variability relative to biological variability through CV-ratio filtering; additionally, QC injections showed highly consistent feature-intensity distributions throughout the analytical sequence, with sample medians remaining close to the overall QC median, supporting good analytical stability and the absence of marked intensity drift across the batch (Supplementary Figure S2). To eliminate background signals originating from the culture media rather than bacterial activity, each sample was subtracted of its corresponding blank. The dual-column strategy and HR-MS detection allowed the identification of 265 metabolites with a good level of confidence (Supplementary Table S2). All the identification levels are indicated in Supplementary Table S2 for each single identified metabolite. The indication of the chromatographic column and the polarity used to identify every single molecule are also reported (BEH POS, RP-C18 POS, RP-C18 NEG). All the parameters and thresholds that justify the metabolites identification are also included according to margherita R package (Mosca et al., 2024). The principal component analysis (PCA) performed on the complete dataset for each chromatographic column [Figure 1 and Supplementary Figure S3, BEH AMIDE POS (A), RP-C18 POS (B), RP-C18 NEG (C)] clearly shows a separation of the samples, indicating distinct metabolomic profiles associated with each fermentation broth. For each metabolite, significant pairwise differences between strain groups, assessed by one-way ANOVA followed by Tukey’s HSD, and the direction of effect (i.e., which group shows higher abundance) are reported in Supplementary Tables S2. Combining the identified metabolites retrieved from BEH AMIDE and RP-C18 analysis in both polarities, 204 metabolites are statistically different in at least one comparison (ANOVA *post-hoc p*-adjusted less than 0.05) (Supplementary Table S2) and are reported in the heatmap shown in Supplementary Figure S4. Unsupervised hierarchical clustering of blank-subtracted metabolomic features (BEH-AMIDE/RP-C18, POS/NEG) resolved clear, strain-dependent patterns. Replicates grouped tightly by condition, with MIC_LAC_SALI and MIC_LAC_PLAN co-clustering and showing higher levels of aromatic amino acids metabolites, mainly tryptophan derivatives, such as indole-3-lactic acid, indole-3-acetic acid, and indole-3-carboxaldehyde. This finding, together with the accumulation of branched-chain amino acids (valine, leucine/isoleucine) and phenolic/phenylpropanoid acids (e.g., p-coumaric, ferulic), is consistent with active amino-acid remodeling and phenolic biotransformation in these two strains. In contrast, MIC_BIFI_BREV formed a distinct branch enriched in nucleotides/nucleosides (AMP, GMP, adenosine, guanosine, and cyclic nucleotides) and ribose-5-phosphate, indicating divergent purine/pyrimidine turnover and energy signaling. MIC_LAC_AMYL separated due to increased central-carbon metabolites, with elevated lactic, pyruvic, and succinic acids and intermediates such as glycerol-3-phosphate, reflecting strong fermentative flux. MIC_LAC_KEFI was the most distinct cluster, marked by higher TCA-cycle intermediates (citric and malic acids, with accompanying organic acids) and selected small bioactives, indicating a different redox/energy balance. Overall, while MIC_LAC_SALI and MIC_LAC_PLAN share a partially overlapping amino-acid/indole/phenolic signature, MIC_BIFI_BREV, MIC_LAC_AMYL, and MIC_LAC_KEFI remain clearly separated by enrichment in nucleotides, core fermentation acids, or TCA intermediates, confirming that each strain imprints a characteristic metabolic profile. Supplementary Table S2 allows to specifically pick the differentially represented metabolites in each of the analyzed strains as well as to select the common metabolites. Pathway enrichment analyses performed with the FELLA package are reported in the Supplementary Tables S6–S17, for human and each bacterial strain.

**Figure 1:**
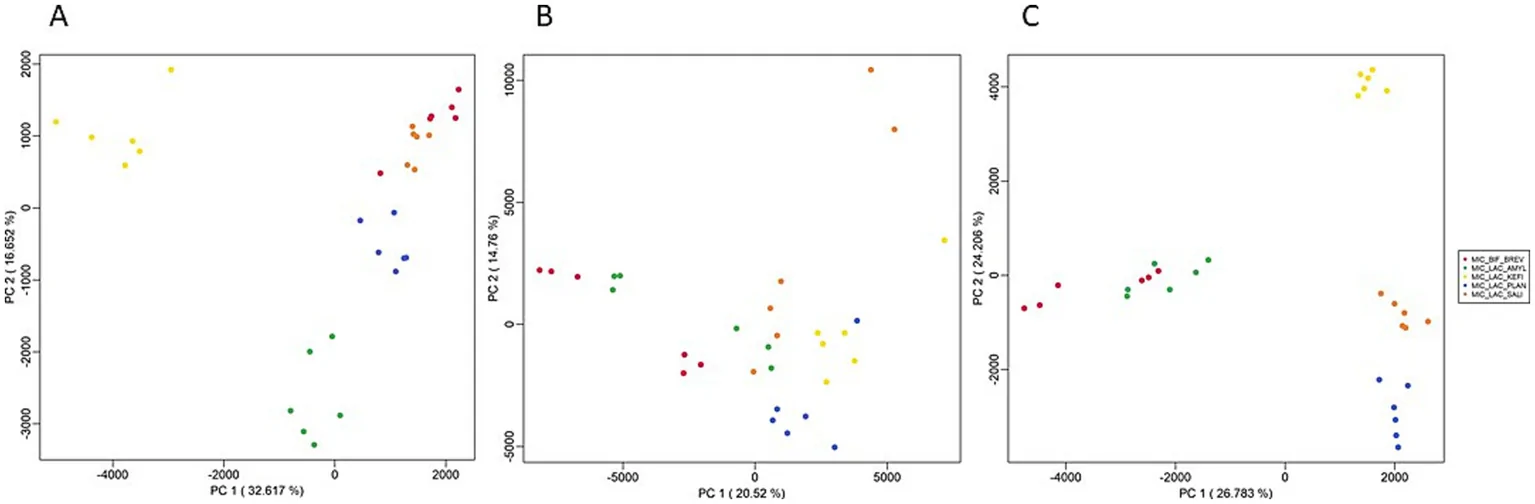
Principal component analysis (PCA) of untargeted metabolomics data derived from the five lactic acid bacterial strains. Every strain was analysed in three technical replicates for each of the two biological replicates. Untargeted metabolomics data are reported for BEH AMIDE column separation in positive acquisition mode **(A)**; RP-C18 column separation in positive **(B)** and negative **(C)** acquisition mode, respectively. Each dot represents one technical replicate of LC–MS injection from two independent biological fermentations; technical replicates are shown for visualization of analytical variability and do not represent independent biological replicates.

#### 3.2.2 Untargeted lipidomics analysis

Lipidomic profiling of the fermentation broths was performed using high-resolution mass spectrometry (HR-MS) coupled with reverse-phase chromatography on a BEH C8 column. This separation method enabled efficient resolution of a wide range of lipid classes, including phospholipids, lysophospholipids, sphingolipids, and neutral lipids such as diacylglycerols (DG) and triglycerides (TG). The use of the C8 stationary phase provided optimal retention and separation of lipids with diverse hydrophobic chain lengths and degrees of unsaturation. Lipid detection was performed in both positive and negative electrospray ionization modes to maximize lipid coverage, yielding identification of 48 lipids in positive mode and 180 in negative mode (Supplementary Table S4). The PCA performed on the complete lipidomic dataset clearly shows a separation of the samples, indicating distinct lipidomic profiles associated with each fermentation broth (Figure 2). Unsupervised hierarchical clustering of the statistically different lipids revealed clear strain-dependent lipid signatures among the five lactic acid bacterial fermentations. A total of 186 unique lipids, significantly altered in at least one comparison (ANOVA, *post-hoc* adjusted *p* < 0.05) (Supplementary Figures S5A,B), were included in the analysis. Several lipid classes were represented, including glycerophospholipids (PC, PE, PI, PS), lysophospholipids (LPC, LPE, LPA, LPG, LPI), neutral lipids (DG, TG), and less common subclasses such as ether lipids, plasmalogens, and sphingolipids. The clustering highlights that each strain generates a characteristic lipid imprint in the fermentation broth, shaped by differential modulation of structural phospholipids, lysophospholipids, neutral lipids, and specialized bioactive classes. Certain phospholipids and lysophospholipids were commonly modulated across multiple strains, indicating partially shared metabolic effects. In contrast, distinct subsets of lipids were specifically enriched in individual strains, highlighting strain-differential pathways of lipid metabolism and transformation.

**Figure 2:**
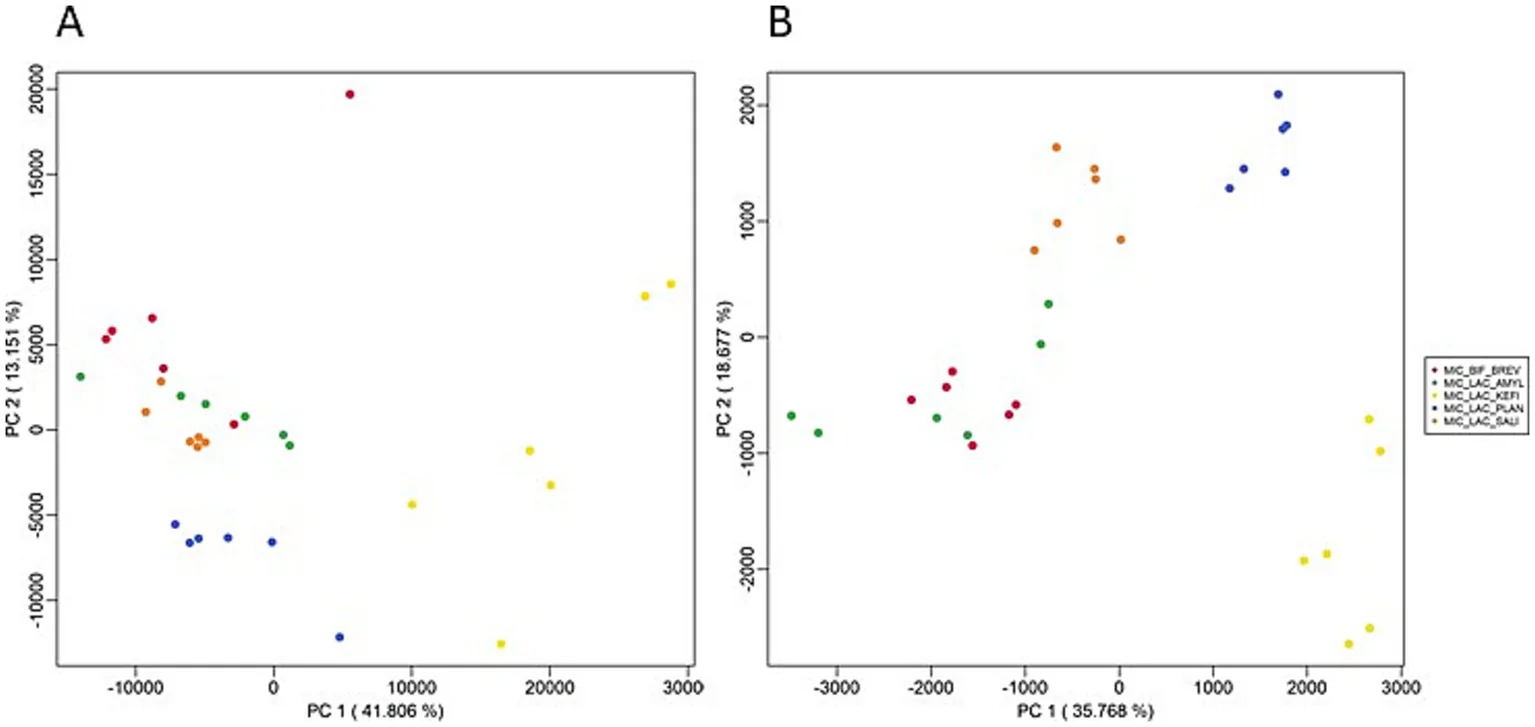
Principal component analysis (PCA) of untargeted lipidomics data derived from the five lactic acid bacterial strains. Every strain was analysed in three technical replicates for each of the two biological replicates. Untargeted lipidomics data are reported for BEH C8 column separation in positive **(A)** and negative **(B)** acquisition mode, respectively. Each dot represents one technical replicate of LC–MS injection from two independent biological fermentations; technical replicates are shown for visualization of analytical variability and do not represent independent biological replicates.

The observed clustering showed that strains producing higher levels of lysophosphatidylcholines (LPC) and lysophosphatidylethanolamines (LPE) clustered together, reflecting a common remodeling of membrane phospholipids through hydrolysis, as in the case of MIC_LAC_SALI and MIC_LAC_PLAN. By contrast, strains characterized by elevated diacylglycerols (DG) and triglycerides (TG), such as MIC_BIFI_BREV, formed a distinct cluster, consistent with differences in neutral lipid metabolism. Another subgroup, represented by MIC_LAC_AMYL, was defined by enrichment in phosphatidylethanolamines (PE) and phosphatidylinositols (PI), which are structural phospholipids important for membrane integrity and signaling. In addition, one strain, specifically MIC_LAC_KEFI, separated uniquely due to an overrepresentation of plasmalogens and ether phospholipids, lipids typically linked to antioxidant protection and specialized cellular functions.

### 3.3 Identification and quantification of 3-phenyllactic acid (PLA) and indole-3-lactic acid (ILA) metabolites by LC–MS/MS

Each strain exhibited a specific metabolomic profile; however, among the most abundant and relevant metabolites identified by untargeted metabolomics, 3-phenyllactic acid (PLA) and Indole-3-lactic acid (ILA) were commonly overrepresented across all strains.

In order to verify and confirm the untargeted metabolomics results, in fermented broths PLA and ILA were quantified by LC–MS/MS technique using deuterium-labeled molecules as internal standards to correct for the metabolite extraction yield (Table 1 and Figure 3). The analytical results are shown in the Table 2, as amount of each metabolite (ng) per volume of broth (μL).

**Table 1:** 3-phenyllactic acid (PLA) and indole-3-lactic acid (ILA) description.

3-phenyllactic acid (PLA) and indole-3-lactic acid (ILA)			
Metabolite	RT (min)	Parent ion *m/z* (M-H)^−^	Fragment ion *m/z* (M-H)^−^
PLA	4.36	165.0557	119.0504
PLA-d3	4.33	168.0745	121.0627
ILA	4.66	204.0665	130.0663
ILA-d5	4.61	209.0981	135.0979

RT (minutes), parent ion and fragment ion, in negative mode, are indicated for PLA and ILA, along with their corresponding deuterium-labeled standards: 3-phenyllactic acid-d_3_ (PLA-d3) and indole-3-lactic acid-d_5_ (ILA-d5).

**Figure 3:**
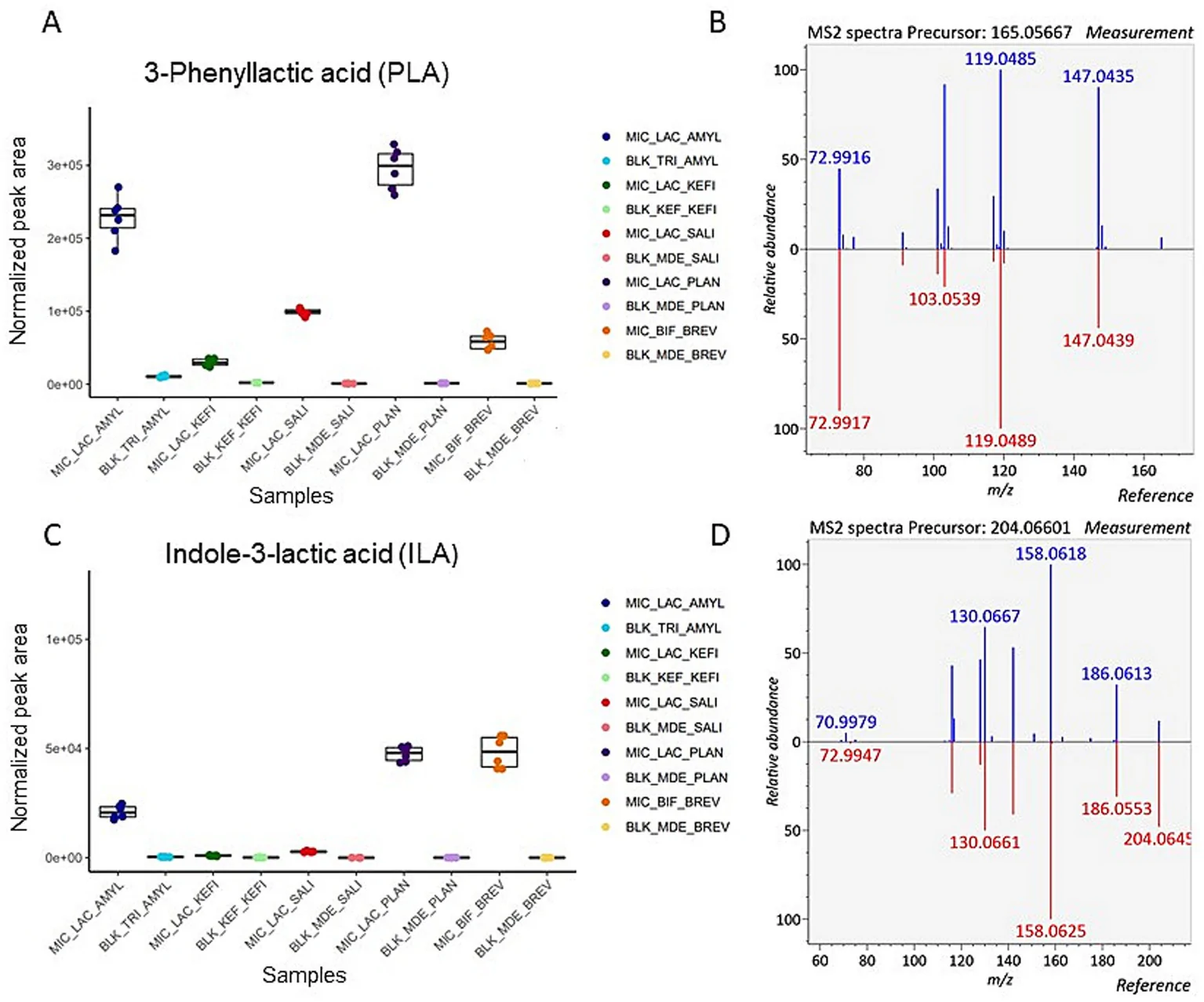
Targeted quantification of PLA and ILA by LC–MS/MS. The box plots show the peak area for PLA **(A)** and ILA **(C)** evaluated in each lactic acid bacterial strain and corresponding blanks. Each sample was analysed in three technical replicates per biological replicate. Each dot represents one technical replicate. The minimum, the first quartile, the median, the third quartile and the maximum are indicated. The MS/MS spectrum of PLA **(B)** and ILA **(D)** are reported, in comparison to the corresponding reference MS/MS spectra. Each dot represents one technical replicate of LC–MS injection from two independent biological fermentations; technical replicates are shown for visualization of analytical variability and do not represent independent biological replicates.

**Table 2:** PLA and ILA quantification for each bacterial strain.

ILA (ng/μL) PLA (ng/μL)					
Name	Code	Average	SD	Average	SD
*L. amylovorus SGL 14*	MIC-LAC-AMYL	0.243	0.036	3.7358	0.8257
*B. breve SGB 01*	MIC-BIF-BREV	0.4847	0.119	0.6256	0.2155
*L. kefiri SGL 13*	MIC-KEFI	0.0478	0.0029	0.3114	0.352
*L. Plantarum SGL 07*	MIC-PLAN	0.4848	0.0352	6.2317	0.2461
*L. Salivarius SGL 03*	MIC-SALI	0.0584	0.0032	1.0834	0.0852

Average values of three technical replicates per biological replicate and relative standard deviation (SD) are reported for each bacterial strain.

Quantitative analysis confirmed the presence of PLA and ILA in the CFS from each strain. The quantities of PLA and ILA varied among strains; however, since they are primary metabolites, differences in metabolism led to different yields. Interestingly, since PLA and ILA are known as potential therapeutic agents having a beneficial effect on human health, they could be used in the formulation of pharmaceuticals and functional foods. For this reason, they were selected as markers for the optimization of the fermentation process. Specifically, since *L. plantarum* SGL 07 showed the highest metabolite yields in the basal medium, it was selected as the reference strain for optimization of fermentation conditions.

### 3.4 Fermentation process optimization for enhanced metabolite production

PLA and ILA concentration was determined by HPLC at different time points during the fermentation process (Figure 4). The results confirmed that these molecules were produced during the exponential growth phase and accumulated during the stationary phase. Maximum biomass concentration was found after 10 h of fermentation (log_10_ 9.5–10.0) remaining stable until 20 h.

**Figure 4:**
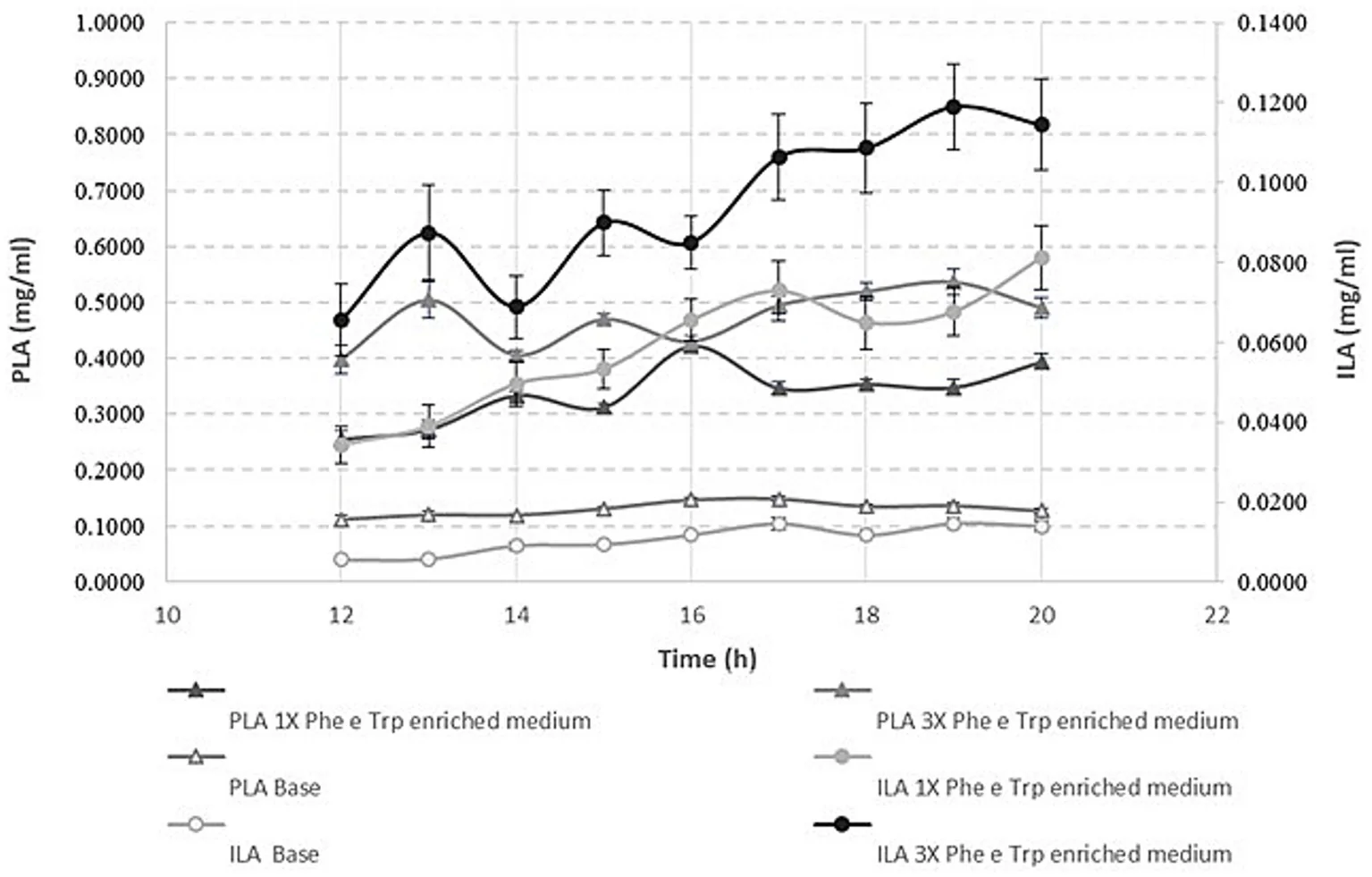
Optimization of fermentation conditions. Production of PLA (triangle indicator) and ILA (circle indicator) by SGL 07 with the addition of tryptophan and phenylalanine compared with the non-supplemented medium.

The PLA concentration measured in the higher supplemented medium was approximately 3.5 fold higher (from 0.15 mg/mL to a 0.54 mg/mL), while ILA showed an increase of about 8 fold (from 0.015 mg/mL to 0.119 mg/mL) compared to the basal medium.

We also investigated the effects of halving the sugar content or substituting some components in the fermentation medium. Specifically, we replaced meat peptones with vegetable peptones, tween 80 with glycerol and fibers. However, none of these modifications affected the yields of PLA and ILA (data not shown).

## 4 Discussion

The untargeted metabolomic and lipidomic characterization of CFS derived from food-grade microorganisms provides insights into the metabolic complexity of fermentation by-products and their potential value as sources of putative postbiotic metabolites. The identification of distinct metabolic signatures in our study highlights how different microorganisms generate distinct chemical repertoires that may interact with host physiological pathways. These findings should be interpreted as an exploratory mapping of metabolite diversity rather than direct evidence of biological activity. The results are discussed below across broader biological domains (Figure 5).

**Figure 5:**
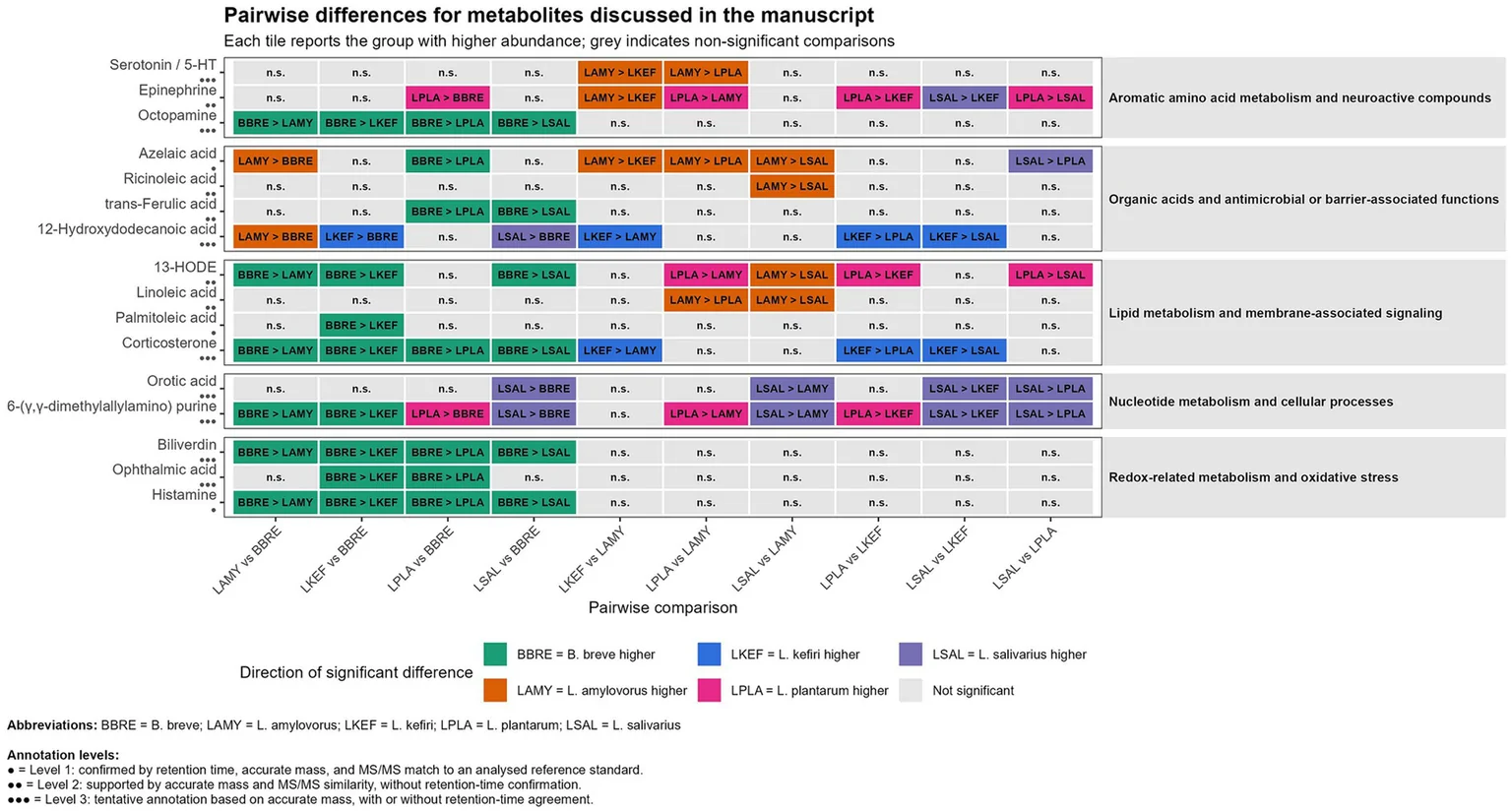
Pairwise comparison of selected metabolites across strains grouped by biological function. This visualization reports an overview of the results considering the metabolites mentioned in the discussion section. Each rectangle represents a pairwise comparison between two strains and indicates the strain with significantly higher abundance of the metabolite. Color coding denotes the direction of the difference according to the legend, while grey tiles (n.s.) indicate non-significant comparisons. Statistical significance was determined as described in the Methods section.

### 4.1 Aromatic amino acid metabolism and neuroactive compounds

Several metabolites linked to aromatic amino acid metabolism and biogenic amine pathways were identified in a strain-dependent manner. In particular, the supernatant of *Lactobacillus amylovorus* SGL 14 contained serotonin (5-HT), a key mediator of gut–brain axis communication involved in intestinal motility and immune signaling (Layunta et al., 2021), and previously associated with *Lactobacillus*-mediated modulation of host serotonin levels (Liu et al., 2023). Neuroactive compounds were also detected in other strains: *Lactiplantibacillus plantarum* SGL 07 showed catecholamine-related metabolites such as epinephrine which can influence the gut–brain axis regulating the brain functions and cognition and affect intestinal motility, vascular tone and local immune signaling potentially contributing to neuromodulatory postbiotic effects (Chen et al., 2021; Montagnani et al., 2023). *Bifidobacterium breve* SGB 01 was associated with octopamine, a biogenic amine, structurally similar to noradrenaline, implicated in host–microbe signaling pathways. In *Caenorhabditis elegans*, for example, bacterially-derived tyramine is converted by the host into octopamine, which then modulates sensory behavior via the OCTR-1 receptor (O'Donnell et al., 2020). Together, these findings suggest that different strains may contribute to neuroactive metabolite pools, although their functional relevance remains to be validated, within the dynamic host-microbiota environment and *in vivo* context.

### 4.2 Organic acids and antimicrobial or barrier-associated functions

Organic acids and phenolic compounds with reported antimicrobial or anti-inflammatory activity were distributed across multiple strains. For instance, *Lactobacillus amylovorus* SGL 14 produced azelaic acid and ricinoleic acid, compounds with known roles in modulating inflammatory pathways and microbial growth (Nisbett et al., 2024; Petrovici et al., 2025). Mechanistically, azelaic acid has been shown to modulate reactive oxygen species, suppress inflammatory pathways, and even upregulate PPAR-γ expression (Shucheng et al., 2024). The production of azelaic acid by *L. amylovorus* may suggests a novel, microbiota-derived source of this bioactive agent, potentially useful for local or systemic inflammatory control. Similarly, *Bifidobacterium breve* SGB 01 and *Lactiplantibacillus plantarum* SGL 07 were associated with phenolic metabolites such as trans-ferulic acid, which has been shown to support intestinal barrier function and reduce inflammatory signaling (Hwang et al., 2022; Huang et al., 2025). In addition, *Lentilactobacillus kefiri* SGL 13 showed the presence of 12-hydroxydodecanoic acid, a hydroxylated fatty acid with antimicrobial activity against bacteria and fungi. This medium-chain hydroxylated fatty acid is found in natural products such as royal jelly and has demonstrated activity against Gram-positive bacteria (*Staphylococcus aureus*, *Bacillus subtilis*) and *Escherichia coli*, as well as *Candida albicans* (Douglas et al., 2025). These patterns suggest that different strains may contribute complementary functions within antimicrobial defense and epithelial support.

### 4.3 Lipid metabolism and membrane-associated signaling

Lipidomics profiling revealed clear strain-dependent differences in lipid composition. *Lactobacillus amylovorus* SGL 14 was characterized by the presence of oxylipins such as 13-HODE, an oxidized lipid derived from linoleic acid, a ligand of PPARγ involved in anti-inflammatory signaling in epithelial and immune cells (Bull et al., 2003). In addition, 13-HODE has been shown to reduce mTOR signaling, thereby suppressing the growth of cancer cells as well as tumor xenografts (Park et al., 2025). *Bifidobacterium breve* SGB 01 exhibited lipids such as palmitoleic acid, a monounsaturated fatty acid (lipokine) known to inhibit NF-κB activation and modulate macrophage polarization (Souza et al., 2017; Chan et al., 2015), with additional evidence supporting its role in intestinal barrier function (Chen et al., 2023; Cetin et al., 2024). Detection of corticosterone-related species (e.g., corticosterone) suggests microbial engagement in steroid transformation; gut bacteria are increasingly recognized for their capacity to biotransform steroid precursors, with possible local consequences for stress- and inflammation-related signaling (Arp et al., 2024). Moreover, lysophospholipids were enriched in selected *Lactobacillus* strains, indicating a potential role in epithelial integrity and inflammatory signaling (He et al., 2025), whereas *Bifidobacterium breve* SGB 01 showed a relative enrichment in neutral lipids such as triacylglycerols, possibly reflecting distinct metabolic strategies (Yamamura et al., 2021). The presence of structural phospholipids and ether lipids across some strains may also evoke a contribution to cellular stress response. In particular, lipids associated with antioxidant protection, such as plasmalogens, reinforce the concept that fermentation-derived lipids may exert cytoprotective effects in the host (Russo et al., 2020). These findings confirm that microbial fermentation broths may represent a relevant source of lipid-based putative postbiotics, extending the functional landscape beyond polar metabolites and highlight that lipid metabolism is an active and strain-distinct component of microbial fermentation.

### 4.4 Nucleotide metabolism and cellular processes

Metabolites involved in nucleotide metabolism were primarily associated with specific strains. *Ligilactobacillus salivarius* SGL 03 was characterized by the presence of orotic acid, an intermediate in pyrimidine biosynthesis linked to epithelial renewal and metabolic regulation (Löffler et al., 2015), as well as purine-related compounds such 6-(γ,γ-dimethylallylamino) purine, cytokinin-like molecule with reported anti-inflammatory properties (Garban and Ilia, 2024; Fathy et al., 2022). These findings suggest a potential contribution of this strain to pathways involved in cellular turnover and metabolic homeostasis, although the biological relevance *in vivo* remains to be established.

### 4.5 Redox-related metabolism and oxidative stress

Markers of redox-related metabolism were also detected in a strain-dependent manner. *Bifidobacterium breve* SGB 01 contained biliverdin, a potent antioxidant capable of modulating NF-κB signaling (Mancuso, 2023), and ophthalmic acid, associated with glutathione metabolism and oxidative stress (Schomakers et al., 2024). In addition, histamine is also detected in the *Bifidobacterium breve* SGB 01 supernatant, and this finding could be linked with the importance of this amino acid in the global immunomodulatory and antioxidant potential seen in *Lactobacillus reuteri* (Gao et al., 2015).

Overall, untargeted metabolomics and lipidomics demonstrate that each strain contributes a distinct subset of metabolites potentially linked to different biological domains, including neuroactive signaling, antimicrobial activity, lipid-mediated regulation, nucleotide metabolism, and redox balance. This reinforces the concept that postbiotic potential is inherently strain-distinct and context-dependent. From an applied perspective, these findings highlight that fermentation media, often considered waste streams, represent a rich and exploitable source of microbial derived metabolites with potential multifunctional applications. Recent metabolomics studies have similarly highlighted the strain-dependent metabolic diversity of postbiotic preparations derived from Bifidobacterium and Lactobacillus species, supporting the value of metabolomic profiling for characterizing microbial-derived bioactive molecules (Liu et al., 2026). However, the present study was based on metabolite annotation through untargeted metabolomics and did not include direct functional validation of the detected compounds or CFS. Therefore, the biological activities discussed should be interpreted as putative and inferred from previously published evidence rather than experimentally demonstrated in the current work. In addition, each bacterial strain was cultured in two independent biological replicates, defined as separate cultures grown. For each biological replicate, samples were analyzed in technical triplicate to ensure analytical reproducibility and instrument stability. Although technical replicates provide important information on measurement precision, they do not account for biological variability. The statistical analyses performed in this study are intended to provide an exploratory comparison of metabolite profiles among strains rather than definitive conclusions and further validation with larger sample sizes is warranted. Moreover, the use of different media formulations and fermentation conditions may contribute to the observed metabolomic and lipidomic differences among strains. The selection of strain-differential cultivation media was necessary to ensure optimal growth and viability for each microorganism, given their distinct physiological requirements. To minimize medium-derived contributions, medium-specific blanks were included and subtracted during data processing. Importantly, the aim of the study was not to attribute every detected metabolite exclusively to intrinsic strain metabolism independent of environmental factors, but rather to characterize the fermentation-associated metabolomic and lipidomic fingerprints generated by each strain within biologically relevant cultivation settings. At the same time, the distinct metabolite profiles and clustering patterns observed under industrially relevant fermentation settings highlight the potential of untargeted metabolomics as a tool to explore and optimize fermentation processes aimed at enriching metabolites of biological interest.

To demonstrate the feasibility of this workflow, the untargeted findings were subsequently investigated using targeted LC–MS/MS and HPLC analyses. Given the limited selectivity of DAD detection at 210 nm, the HPLC-DAD measurements should be interpreted with appropriate caution. The resulting values were primarily used to assess relative differences in PLA and ILA levels among fermentation supernatants and should not be considered equivalent to structure-confirmed LC–MS/MS quantification. Accordingly, the HPLC-DAD data provide supportive quantitative information, while the LC–MS/MS analysis using authentic standards and characteristic MS/MS fragmentation provides stronger evidence for metabolite identity. Although PLA and ILA quantification was supported by matrix-matched calibration curves and isotope-labelled internal standards, a comprehensive analytical validation was not performed; therefore, these measurements should be considered supportive quantitative data rather than fully validated quantitative determinations. This integrated analytical strategy confirmed the presence and relative enrichment of PLA and ILA across the strains investigated. By transitioning from exploratory screening to a targeted analytical platform, we were able to further characterize these metabolites and assess their production under the tested fermentation conditions. Consequently, the optimized fermentation conditions increased PLA and ILA production by 3.5-fold and 8-fold, respectively, under the tested experimental conditions. These results highlight the value of combining untargeted and targeted workflows to identify and further characterize metabolites of potential biological relevance in microbial fermentations. Nonetheless, this study underscores the value of leveraging strain-distinct metabolic outputs to support the future development of targeted postbiotic strategies, which may be considered as complementary or adjunctive approaches to conventional interventions. In this way, this report contributes to the broader concept of valorizing microbial bioprocessing streams as sustainable reservoirs of biologically relevant molecules.

Despite their promising bioactive potential, postbiotics also present important limitations when considered as stand-alone interventions. Unlike live microorganisms, postbiotics do not actively colonize the host or dynamically adapt to the intestinal environment, which may limit their long-term efficacy and persistence of action (Scott et al., 2022; Salminen et al., 2021). In addition, although generally regarded as safer and more stable than probiotics, the biological effects of complex metabolite mixtures remain incompletely understood, and potential off-target or context-dependent effects cannot be excluded (Aguilar-Toalá et al., 2018; Żółkiewicz et al., 2020). Another important consideration is the loss of ecological complexity associated with the use of isolated metabolites or CFS. In the gut ecosystem, microbial functionality is strongly influenced by interspecies cross-feeding interactions and bidirectional host-microbe signaling networks (Wegh et al., 2019), which may not be fully recapitulated by postbiotic preparations alone.

Altogether, these findings support the emerging interest in postbiotic-inspired approaches bridging fermentation biotechnology and host-oriented microbiome research. Further mechanistic and *in vivo* studies will be essential to define physiological relevance, optimize site-specific delivery strategies (including dose, timing, off targets) and translate these bioactive compounds into next-generation functional ingredients or microbiome-inspired therapeutics.

## Data Availability

The R codes written to perform the data elaboration are reported in the GitHub repository: https://github.com/FrigerioGianfranco/UntargetedMetabolomics_FoodGradeMicroorganisms_Fermentation. The raw files, and feature and input tables for the data elaboration, are reported in the Zenodo repository: https://doi.org/10.5281/zenodo.20444054.

## Footnotes

1
https://www.reifycs.com/AbfConverter

## Supplementary material

SUPPLEMENTARY FIGURE S1 Calibration curves for targeted quantification of PLA and ILA by LC-MS/MS. 3-Phenyllactic acid (PLA) **(A,B)** and Indole-3-lactic acid (ILA) **(C,D)** were used at different concentrations in blank media (to mimic matrix effect) to calculate the linear equation and *R*^2^ value. Each value of peak area was corrected for the area of the corresponding internal standard (IS), as for the samples. IS: 3-Phenyllactic acid-d_3_ (PLA-d3) and Indole-3-lactic acid-d_5_ (ILA-d5).

SUPPLEMENTARY FIGURE S2 Variation of feature intensities across pooled quality-control (QC) samples in HILIC POS **(A)**, RP-C18 NEG **(B)**, and RP-C18 POS **(C)** analyses. Feature intensities were normalised to the feature-specific median across QCs and log2-transformed. Boxplots show the distribution of relative intensities across all features for each QC injection, ordered by analytical sequence; the line connects sample medians, and the dashed line indicates no deviation from the feature-specific median.

SUPPLEMENTARY FIGURE S3 Principal component analysis (PCA) of HILIC POS **(A)**, RP-C18 NEG **(B)**, and RP-C18 POS **(C)** analyses, including the quality-control (QC) samples. The clear clustering of QC samples demonstrates good analytical reproducibility and stability, indicating that technical variability was limited relative to the biological variability observed among study samples.

SUPPLEMENTARY FIGURE S4 Unsupervised hierarchical clustering of statistically different metabolites derived from the five bacterial strains. 204 metabolites, which were statistically different in at least one comparison (ANOVA *post-hoc* adjusted *p*-value < 0.05) were used to construct the heatmap. The up (pink) and down (light blue) represented metabolites are partly in common among the different fermentation broths, while some metabolites were specifically overrepresented in each strain. Each metabolite is reported along with the indication of the separation method (BEH AMIDE or RP-C18) and polarity (POS or NEG) used for its detection. Each column represents one technical replicate per strain.

SUPPLEMENTARY FIGURE S5 Unsupervised hierarchical clustering of statistically different lipids derived from the five bacterial strains. 186 lipids (depicted in two heatmaps S5A and S5B), which were statistically different in at least one comparison (ANOVA *post-hoc* adjusted *p*-value < 0.05) were used to construct the heatmap. The up (pink) and down (light blue) represented lipids are partly in common among the different fermentation broths, while some lipids were specifically overrepresented in each strain. Each lipid is reported along with the indication of the separation method (BEH RP-C8) and polarity (POS or NEG) used for its detection. Lipids are grouped according to their lipid class. Each column represents one technical replicate per strain.
